# Metabolic Dysfunction-Associated Steatotic Liver Disease and Emerging Oligonucleotide Therapies

**DOI:** 10.7150/ijbs.127996

**Published:** 2026-02-11

**Authors:** Qionghui Chen, Jing Jiang, Zhixin Chiang, Xiangyu Li, LiWen Zhang, Lian Wang, Hung-fat Tse, Shihua Wang, Qizhou Lian

**Affiliations:** 1 State Key Laboratory of Ecological Pest Control for Fujian and Taiwan Crops, Fujian Key Laboratory of Pathogenic Fungi and Mycotoxins, School of Life Sciences, Fujian Agriculture and Forestry University, Fuzhou, 350002, China.; 2 Faculty of Synthetic Biology, Shenzhen University of Advanced Technology, Shenzhen, 518107, China.; 3 State Key Laboratory of Quantitative Synthetic Biology, Shenzhen Institute of Synthetic Biology, Shenzhen Institutes of Advanced Technology, Chinese Academy of Sciences, Shenzhen, 518055, China.; 4 Center for Translational Stem Cell Biology, Hong Kong, and State Key Laboratory of Pharmaceutical Biotechnology, The University of Hong Kong, Hong Kong SAR, China.; 5 Prenatal Diagnostic Center and Cord Blood Bank, Guangzhou Women and Children's Medical Centre, Guangzhou Medical University, Guangzhou, China.; 6 Department of Medicine, HKUMed Laboratory of Cellular Therapeutics, University of Hong Kong, HongKong, China.; 7 State Key Laboratory of Food Nutrition and Safety, Key Laboratory of Industrial Fermentation Microbiology of the Ministry of Education, Tianjin Key Laboratory of Industrial Microbiology, College of Biotechnology, Tianjin University of Science and Technology, Tianjin, 300457, China.

**Keywords:** Metabolic Dysfunction-Associated Steatotic Liver Disease, Oligonucleotide, delivery systems, therapeutic targets

## Abstract

Metabolic Dysfunction-Associated Steatotic Liver Disease (MASLD) is a prevalent chronic liver condition characterized by pathological fat accumulation in hepatocytes, with a global prevalence of approximately 30% that continues to rise. Current treatment options are limited, highlighting an urgent need for novel therapeutic strategies. This review systematically examines the emerging promise of oligonucleotide-based drugs for MASLD treatment, including antisense oligonucleotide (ASO), small interfering RNA (siRNA), microRNA (miRNA) mimic or inhibitor, small activating RNA (saRNA) and splicing-switching oligonucleotide (SSO). We summarize the mechanisms of action of these therapeutics, which enable precise targeting of genes involved in MASLD pathogenesis. Furthermore, the review explores advanced delivery systems, particularly N-acetylgalactosamine (GalNAc) conjugation, which enhances hepatocyte-specific targeting. Finally, we discuss the current challenges facing oligonucleotide drug development and outline future directions for this rapidly advancing field, underscoring its potential to revolutionize MASLD management.

## Introduction

Metabolic Dysfunction-Associated Steatotic Liver Disease (MASLD) encompasses a wide spectrum of liver conditions, ranging from simple steatosis (MASL) to more severe forms such as metabolic dysfunction-associated steatohepatitis (MASH), end-stage cirrhosis, and even liver cancer^1^-^4^. MASLD is predominantly hallmarked by lipid deposition in hepatocytes, which is often accompanied by systemic metabolic disturbances, including insulin resistance and dysregulated carbohydrate and lipid metabolism. MASLD is typically attributed to a complex interplay of metabolic and pathological alterations, including genetic susceptibility, lipid metabolism abnormalities, oxidative stress, lipid toxicity, mitochondrial dysfunction, inflammation, dysbiosis of gut microbiota, and endoplasmic reticulum (ER) stress^4^, ^5^. This complex pathological mechanism undoubtedly poses a huge challenge for single-action drug development. Currently, several potential drugs for the treatment of MASLD are in phase II or III clinical trials^6^, acting on multiple targets, such as C-C motif chemokine receptor 2/5^7^, peroxisome proliferator activated receptors α/β^8^, farnesoid X receptor, and apoptosis signaling kinase 1^9^. However, due to their safety and efficacy and the complicated pathogenesis of MASLD, decades of target exploration and hundreds of clinical trials have failed, although the first drug, resmetirom, has recently been approved for the management of MASH^10^-^12^.Therefore, there is a need for new drug candidates, especially innovative candidates with new targets.

Oligonucleotides are short nucleic acid polymers that have emerged as a powerful therapeutic modality for treating a wide range of diseases. These chemically modified analogs, typically around 20 nucleotides in length^13^, function by selectively binding to target mRNA via Watson-Crick base pairing, thereby controlling the production of specific proteins. This mechanism allows oligonucleotide therapies to address the underlying causes of disease, provide long-lasting effects, and target conditions previously considered intractable^14^, ^15^.

A key advantage of oligonucleotides is that promising lead sequences can be rationally designed based on genomic knowledge. Their pharmacokinetic and pharmacodynamic properties are further optimized through chemical modifications to the nucleobase, ribose sugar, and phosphate backbone, often in conjunction with advanced drug-delivery vehicles^16^-^19^. Substantial investment over the past decade is fueling a rapid transition for this drug class from treatments for rare diseases to therapies for common disorders. This is reflected in a robust market trajectory, with the global oligonucleotide therapeutics market projected to grow significantly, driven by rising approvals and advancements in delivery technologies. For instance, Novartis's acquisition of inclisiran, a cholesterol-lowering therapy for atherosclerotic cardiovascular disease, exemplifies this expansion toward addressing widespread conditions that affect hundreds of millions globally^20^.

This review explores the latest breakthroughs in oligonucleotide therapy for MASLD, emphasizing innovative delivery strategies. It will systematically cover oligonucleotide types, chemical modifications, and advanced delivery systems like GalNAc conjugation for hepatocyte targeting. The discussion extends to clinical applications, focusing on specific MASLD molecular targets, and evaluates the safety profiles of these promising drugs, highlighting their transformative potential for developing clinically viable MASLD therapies.

## Oligonucleotide drug-development strategies for liver disease

As small synthetic nucleic acid polymers, oligonucleotides target messenger RNA (mRNA), non-coding RNA (ncRNA), or DNA via complementary base pairing while also interacting with certain proteins through three-dimensional binding to show their potent gene-silencing capacity. Currently, antisense oligonucleotides (ASOs), small interfering RNAs (siRNAs), microRNA (miRNA) mimics or inhibitors, small activating RNAs (saRNAs) and splicing-switching oligonucleotides (SSOs) are the most intensively studied oligonucleotide species, with diversified action modes, including expression inhibition or activation of functional genes and non-coding transcripts, as well as mRNA splicing modulation^21^.

Oligonucleotides are nucleic acid polymers with the potential to treat or manage a wide range of diseases. In addition to their ability to recognize specific target sequences via complementary base pairing, nucleic acids can also interact with proteins through the formation of three-dimensional secondary structures-a property that is also being exploited therapeutically. Here, we classify oligonucleotides into various types based on their mechanism of action.

### ASO

ASOs are short nucleotide sequences that can induce gene silencing. Typically ranging from 15 to 22 nucleotides in length, ASOs are specifically designed to be complementary to the target RNA in a reverse orientation, which is why they are termed "antisense" oligonucleotides. Through Watson-Crick base pairing, ASOs hybridize with complementary sequences in the target mRNA, thereby suppressing gene expression. These straightforward base pairing rules govern the interaction between antisense oligonucleotides and their targets, enabling the design of oligonucleotides to target virtually any gene with a known sequence^22^.

**Figure 2:**
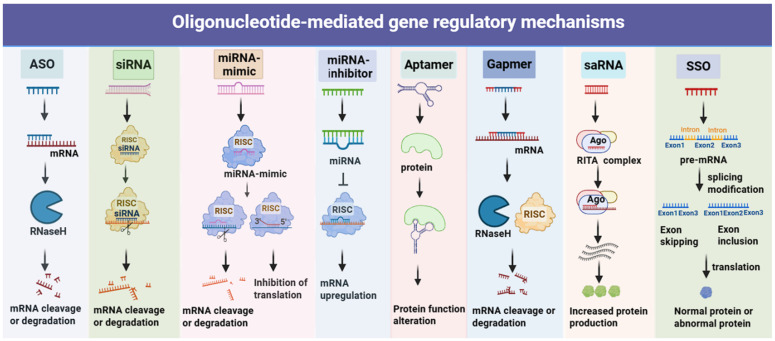
** Mechanisms of action for oligonucleotides.** Representative mechanisms of action and intracellular localisation for (1) Antisense Oligonucleotide (ASO): Binds target mRNA (nucleus/cytoplasm); inhibits translation or recruits RNase H to cleave RNA-DNA duplex, reducing target protein. (2) siRNA: Enters cytoplasm; processed by Dicer to form RISC; guide strand cleaves target mRNA for RNAi-dependent silencing. (3) miRNA Mimic: Cytoplasmic; mimics endogenous miRNA, loads into RISC, recognizes multiple target mRNAs, inhibits translation (or mild degradation) to regulate gene networks. (4) miRNA inhibitor: Cytoplasmic antagonist; binds active miRNA with high affinity, blocks mRNA interaction to reverse silencing. (5) Aptamer: A single-stranded oligonucleotide folding into a specific 3D structure; localizing to the cell surface (binding membrane proteins) or cytoplasm (binding soluble proteins), and acting by blocking protein-ligand interactions, inhibiting protein enzymatic activity, or mediating targeted delivery. (6) Gapmer: Chimeric ASO binds mRNA (nucleus/cytoplasm), recruits RNase H to cleave for efficient silencing. (7) saRNA: Localizes to nucleus; binds gene promoters/enhancers, recruits co-activators (Ago2), induces chromatin remodeling to promote transcription. (8) SSO: Specifically binds pre-mRNA, modulates exon skipping/inclusion, and produces functionally normal/abnormal proteins.

ASOs are theoretically designed to regulate the transfer of genetic information to proteins specifically, but the mechanisms by which ASOs induce biological effects are subtle and complex (Fig. 2). Based on the mechanism of action, three major classes of ASOs can be discerned: (a) Downregulation mechanism of degradation. The ASO-mRNA double strands as a substrate recruit RNase H1, leading to degradation of the target transcript. (b) Downregulation mechanism of steric blockage. ASOs bind to pre-mRNA to alter polyadenylation position and decrease mRNA stability and levels. (c) Upregulation mechanism of steric blockage. ASOs inhibit miRNA function to increase the expression of their target mRNA^23^. Thanks to their single-nucleotide precision, ASOs have already redefined the clinical landscape for disorders such as spinal muscular atrophy and inherited retinal dystrophies, converting once-incurable genetic diseases into treatable conditions^24^-^27^.

### siRNA

Small interfering RNA (siRNA) is a type of double-stranded RNA molecule with a molecular weight of approximately 13 kDa. It mediates gene silencing primarily through Watson-Crick base pairing, with its mechanism of action relies on the RNA-induced silencing complex (RISC). Upon binding to complementary sequences in target mRNA, the Argonaute family proteins (such as Ago2) within RISC catalyze the cleavage and degradation of the target mRNA. Alternatively, other Argonaute proteins such as Ago1, Ago3, and Ago4 can direct the mRNA to processing bodies ((P)-bodies) to mediate non-specific degradation^28^.

Compared to small-molecule drugs and monoclonal antibodies, siRNA offer several significant advantages. It does not require recognition of the complex spatial conformation of proteins; instead, it can specifically target any protein-coding gene with a known sequence through base pairing. Consequently, siRNA has the potential to target a broad spectrum of therapeutic genes, including nearly all protein targets of interest^29^.

### miRNA mimic

miRNA mimics simulate endogenous mature miRNAs and achieve gene silencing by enhancing their function. The miRNA mimic binds to the 3' untranslated region (UTR) of the target mRNA, which in turn induces mRNA degradation or translational repression. This study systematically defined the design principles of miRNA mimics for the first time, highlighting that they simulate the function of endogenous miRNAs through chemically synthesized double-stranded RNA and achieve gene silencing by targeting the 3' UTR of mRNA^30^

### miRNA inhibitor

miRNA inhibitors can specifically inhibit the function of endogenous miRNAs, thereby releasing the regulation of target genes by these miRNAs. MiRNA inhibitors work by binding complementarily to mature miRNAs, thereby blocking their interaction with target mRNAs. Types of miRNA inhibitors include antisense oligonucleotides (ASO), Tough Decoy (TuD), and competitive endogenous RNA (ceRNA). These inhibitors function through a mechanism of single-stranded complementary binding to mature miRNAs, thereby blocking their activity^31^.

### Aptamer

Aptamers are single-stranded deoxyribonucleic acid (DNA) or ribonucleic acid (RNA) oligonucleotides (20-100 nucleotides) that adopt three-dimensional structures, enabling them to bind very specifically to protein target sites^32^.

In addition to high-affinity binding to their targets, aptamers also bind with high specificity, much like small molecules, discriminating between target proteins that share similar structural epitopes^33^, ^34^.

### Gapmer

Chimeric antisense oligonucleotides (ASOs) that contain a central block of DNA nucleotides, flanked by modified sequences, usually containing 2′-O-modified or locked nucleic acid (LNA) chemistries. Gapmers are used for gene silencing by stimulating RNA cleavage through the recruitment of RNase H^33^, ^35^.

Research has indicated that the silencing effect of gapmer oligos is not solely dependent on RNase H1 activation; instead, it appears that the RNA-induced silencing complex (RISC) is partially involved^36^.

### saRNA

saRNAs is a group of small double strand RNA, which could effectively activate gene in a sequence specific manner^37^. The mechanism of action of saRNAs is illustrated as follows: (a) Through base complementary pairing, saRNAs specifically bind to either the DNA in the promoter region of target genes or the promoter-associated non-coding RNAs transcribed from these regions. (b) saRNAs associate with Argonaute (Ago) proteins and further assemble into the RNA-induced transcriptional activation (RITA) complex. (c) Guided by the RITA complex, relevant effector components induce epigenetic modifications to the chromatin within the target gene promoter region. (d) The RITA cooperates with RNA polymerase II (Pol II) to initiate target gene transcription, leading to robust upregulation of corresponding mRNA and protein expression^38^.

### SSO

SSOs are a class of nucleic acid drugs targeting the splicing process of precursor mRNA (pre-mRNA). As synthetic short-chain oligonucleotides (typically 15-25 nt), they specifically bind to key splicing elements of pre-mRNA, interfering with the assembly or recognition of the spliceosome, thereby "reprogramming" gene splicing patterns.

Essentially, their mechanism involves occupying binding sites of splicing elements or altering the secondary structure of pre-mRNA, which disrupts the recognition and binding of splicing factors, leading to altered splicing patterns. Examples include exon skipping (i.e., exclusion of specific exons from mRNA) and exon inclusion (i.e., retention of previously silenced exons in mRNA)^39^.

## Modifications of synthetic oligonucleotides

Chemical modifications of synthetic oligonucleotides are essential strategies to enhance their specificity and efficacy for targeting tissues.

### ASO

ASOs modification technology is a therapeutic approach that uses chemically modified single-stranded DNA or RNA molecules to specifically bind to endogenous mRNA targets, thereby regulating gene expression, splicing, and inhibiting microRNA function. Fomivirsen was the first antisense oligonucleotide drug approved for human therapeutic use^29^, ^50^.

The structural modifications of synthetic oligonucleotides (ASO) have undergone three generations of iteration, significantly enhancing the performance of these drugs.

First Generation: The first generation involved replacing the phosphodiester bond with a phosphorothioate (PS) linkage, which enhanced nuclease resistance and extended serum half-life. However, this modification could reduce target binding affinity and induce toxicity. For example, Mipomersen was withdrawn from the market due to hepatotoxicity^51^.

Second Generation: Building on the PS backbone, the second generation introduced 2'-O-methyl or 2'-O-methoxyethyl (2'-MOE) modifications. By optimizing the sugar ring structure, these modifications increased binding affinity and reduced off-target effects. A typical example is Nusinersen, which enhanced splicing regulation through 2'-MOE modification.

Third Generation: The third generation adopted rigid bicyclic structures, such as locked nucleic acid (LNA), cyclohexenyl (cEt), or ethylene-bridged nucleic acid (ENA). These modifications further improved binding affinity and tissue penetration^52^. For example, ASOs modified with LNA have been used in the treatment of liver fibrosis.

### siRNA

The chemical modifications of siRNA, through alterations in the ribose, phosphate, and base groups, have become a key technology for the treatment of MASLD. Ribose modifications (such as 2'-O-methyl (2'-O-Me) and Locked Nucleic Acid (LNA)) enhance target binding affinity and nuclease resistance. Phosphate modifications (such as phosphorothioate (PS)) extend serum half-life to 24-48 hours and promote cellular uptake. Base modifications (such as pseudouridine) reduce immunogenicity^53^.

In MASLD, GalNAc-conjugated siRNA modifications target hepatic pathogenic genes. For example, siRNA targeting PLIN2 (with 2'-MOE/PS modifications) reduces hepatic steatosis and fibrosis. Alnylam's HSD17B13-siRNA, delivered via GalNAc, improves liver function. Preclinical studies have shown that a single injection can safely provide long-lasting inhibition of the target gene. Arrowhead's ARO-HSD has entered Phase I clinical trials, verifying its tolerability and efficacy. In the future, siRNA modification technologies are expected to enable precise modulation of lipid metabolism, inflammation, and fibrosis pathways implicated in MASLD pathogenesis.

### miRNA mimic or inhibitor

The modifications of miRNA mimics or inhibitors include 2'-O-Me and Locked Nucleic Acid (LNA) modifications. The 2'-O-Me modification enhances resistance to ribonucleases in serum and extends the half-life by methylating the 2'-hydroxyl group of the ribose. The first reported 2'-O-Me modified miRNA mimic demonstrated stable intracellular expression and effective gene silencing by targeting the 3' UTR of mRNA^53^, ^54^.

The LNA modification, through its rigid bicyclic structure, increases binding affinity to the target miRNA and reduces off-target effects. Miravirsen, an LNA-modified oligonucleotide targeting miR-122, has entered Phase II clinical trials for the treatment of HCV. The reduction in viral load was found to be dose-dependent on the inhibition of miR-122^55^, ^56^.

## Hepatic delivery systems of oligonucleotides

The liver delivery systems for oligonucleotides are key technologies for enhancing the targeting efficiency and therapeutic efficacy of oligonucleotide drugs in liver tissue. Here are several common delivery systems.

### Cationic liposomes

Cationic liposomes carry a positive charge on their surface, which can electrostatically interact with the phosphate groups of nucleic acids to encapsulate siRNA molecules, forming siRNA-liposome complexes. These complexes can also be adsorbed by negatively charged cell membranes and subsequently enter cells through fusion or endocytosis. Liposome-mediated transfection is currently the most used transfection method, with major commercial products including Lip2000^57^, Lip3000^58^, and LipMAX. The market also offers a wide variety of transfection reagents, including those from Promega (FUGENE), Polyplus, and Lipo8000, among others.

### Lipid nanoparticles

Lipid nanoparticles (LNPs) serve as the core delivery vehicles for siRNA^59^, ^60^. Through the synergistic action of ionizable lipids, cholesterol, phospholipids, and polyethylene glycol (PEG), LNPs effectively address the challenges such as siRNA charge exposure, enzymatic degradation, and endosomal escape. The ionizable lipids become protonated in acidic environments, forming complexes with siRNA that help protect its stability^61^. Cholesterol and phospholipids enhance the structure rigidity of the nanoparticles, while PEG modification reduces non-specific clearance through steric hindrance^62^.

In MASLD, LNP-based delivery systems have demonstrated precise targeting capabilities and therapeutic potential. For hepatic fibrosis, studies using MASH mouse models have observed significant upregulation of IL-11 in activated hepatic stellate cells (aHSCs). By loading siRNA targeting IL-11 into LNPs, the IL-11/ERK signaling pathway is specifically blocked, thereby inhibiting the transdifferentiation of aHSCs and collagen deposition. For lipid metabolism disorders, GalNAc-conjugated LNPs can target the ASGPR receptor on the surface of hepatocytes, efficiently delivering siRNA to silence the MCJ gene. This approach has been shown to reduce hepatic lipid accumulation and alleviate fibrosis in various MASLD models^2^, ^33^, ^63^. With their mature delivery mechanisms and disease-specific targeting strategies, LNPs provide an innovative platform for the gene therapy of MASLD, combining safety and efficacy.

### GalNAc conjugates

N-Acetylgalactosamine (GalNAc), a high-affinity ligand for the asialoglycoprotein receptor (ASGPR), has been conjugated to oligonucleotides through a trivalent cluster structure to achieve precise hepatic targeting and delivery^64^-^66^. This technology, originating from the targeting concept in 1987, has been continuously optimized over nearly two decades and has become a core delivery tool for oligonucleotide drugs such as siRNA, ASO, and Anti-miRNA^15^.

In the therapeutic field of Anti-miRNA, RG-101 from Regulus Therapeutics targets miR-122 through GalNAc conjugation and significantly reduces the viral load in the treatment of hepatitis C 53. In the development of ASO, Ionis Pharmaceuticals' Ligand Conjugated Antisense (LICA) technology, which utilizes GalNAc conjugation, has enhanced drug potency by 10-fold^67^.

The GalNAc conjugation technology, which leverages highly efficient endocytosis mediated by ASGPR (with a 300-fold increase in liver enrichment efficiency), low immunogenicity, and compatibility with various oligonucleotide modalities, has propelled multiple drugs (such as Givosiran and Inclisiran) to market approval and has become a key platform for the gene therapy of liver diseases^15^, ^68^.

### Viral vector platforms

Since the first clinical application of retroviruses in treating severe combined immunodeficiency caused by adenosine deaminase (ADA) deficiency in 1990, lentiviruses (LVs), adenoviruses (AdVs), and adeno-associated viruses (AAV) have become the three major viral vectors used for nucleic acid drug delivery. In the treatment of metabolic diseases, these viral vectors have demonstrated significant potential^69^.

AAV vectors, regulated by inhalation of muscone to control the expression of hFGF21, achieved long-term treatment lasting up to 28 weeks in MASH mouse models, significantly reducing hepatic steatosis and inflammation. Lentivirus vectors delivering siRNA targeting SREBP-1c reduced hepatic SREBP-1c mRNA levels by 60% and triglycerides by 35% in high-fat diet (HFD) mice, effectively inhibiting lipid synthesis^70^. Adenovirus vectors mediating the overexpression of the Nrf2 gene activated the antioxidant pathway, decreased hepatic reactive oxygen species (ROS) levels, and reduced ALT/AST levels by 50% in metabolic steatotic liver disease (MASLD) model mice, significantly improving insulin resistance^71^-^73^.

### Exosomes

The natural exosome consists of a lipid bilayer membrane and an internal lumen. The functional molecules loaded in the lumen, such as nucleic acids (siRNA/mRNA), small-molecule drugs (chemotherapeutic agents), and proteins (antibodies/enzymes), are the core for exerting therapeutic effects. As endogenous extracellular vesicles, exosomes have a diameter comparable to that of nanoscale carriers^74^. Moreover, exosomes can carry various signaling molecules (such as RNA and proteins), which endows them with the potential to serve as drug delivery vehicles. Compared to exogenous nanocarriers, exosomes offer notable advantages such as minimal immunogenicity and reduced toxicity. For example, exosome-carried miR-100-5p can significantly alleviate liver fibrosis in mice induced by a high-fat diet (HFD) by inhibiting the TGF-β/Smad3 signaling pathway in hepatic stellate cells (HSCs)^75^-^78^.

### Antibody-drug conjugates

Antibody's precise targeting to guide siRNA to specific cells, where siRNA silences disease-causing genes, achieving "targeted gene silencing". The mechanism of action of antibody-drug conjugate (ADC) delivery systems is based on a “five-step precision strike”: circulation in the bloodstream, targeted binding, endocytosis, linker cleavage, and drug action^79^, ^80^. This mechanism enables “precision targeting” and “dual therapeutic effects” through antibody navigation, intelligent release, and synergistic killing. For example, CD36 ADC conjugated with a PPARγ agonist (such as rosiglitazone) can inhibit fatty acid uptake and reduce hepatic triglyceride (TG) content by 30%^81^.

## Oligonucleotide in MASLD

Currently, oligonucleotides have been used in the treatment of metabolic-associated steatotic liver disease. These oligonucleotides can target relevant targets and regulate the processes involved in the pathogenesis and progression of MASLD.

### ASO-based therapies

**PNPLA3.** PNPLA3 catalyzes the hydrolysis of triglycerides and the transfer of polyunsaturated fatty acids, thereby mediating the remodeling of phospholipids in hepatic lipid droplets. Genome-wide association studies (GWAS) have identified a strong correlation between PNPLA3 variants and MASLD^91^, ^92^; these variants drive triglyceride retention and the formation of polyunsaturated fatty acid-enriched lipid droplets, which may elevate the risk of MASH and hepatocellular carcinoma. Preclinical studies have shown that PNPLA3-targeting ASOs can alleviate liver inflammation and fibrosis in mouse models.

Currently, AstraZeneca's PNPLA3-targeted ASO therapy (AZD2693) is under Phase I clinical evaluation^93^, ^94^. 2025-published data from these trials report 89% hepatic PNPLA3 mRNA knockdown (biopsy-assessed) in treated subjects; in the 148M homozygous cohort (the MASLD-susceptible variant), early safety signals align with ASO class effects (e.g., mild injection-site reactions), with no dose-limiting toxicities observed.

**DGAT2.** DGAT2 is one of the two isoenzymes that catalyze the synthesis of triglycerides. Along with DGAT1, it is responsible for nearly all triglyceride synthesis. Since the synthesis of triglycerides in MASH is primarily driven by the catalytic activity of DGAT2, inhibiting its activity may suppress triglyceride synthesis and slow the progression of MASLD^95^. Currently, the ASO therapy targeting DGAT2, ION224, has entered Phase II clinical trials for the treatment of patients with confirmed MASH, with the primary endpoint data expected to be available in September of this year^96^, ^97^.

**ANGPTL4.** ANGPTL4 is highly expressed in adipose tissue and the liver, and its dysfunction is closely associated with various metabolic diseases. Studies have shown that loss-of-function variants of ANGPTL4 are significantly correlated with reduced risks of dyslipidemia, coronary artery disease (CAD), type 2 diabetes (T2D), steatosis, metabolic dysfunction-associated steatohepatitis (MASH), and chronic kidney disease (CKD)^98^-^100^. Mechanistically, inactivation of ANGPTL4 can lower LDL-C and triglyceride (TG) levels, inhibit hepatic steatosis and fibrosis, and slow the progression of CAD, T2D, and CKD. In high-fat diet (HFD) mouse models, antisense oligonucleotide (ASO) therapy targeting ANGPTL4 can specifically silence its expression, improve dysmetabolism of glucose and lipids, and alleviate hepatic steatosis and inflammatory responses^29^. These findings provide preclinical evidence supporting ANGPTL4 as a therapeutic target for metabolic diseases, and ASO therapy holds promise as a new strategy for intervening in dyslipidemia and related complications.

**APOC3.** Apolipoprotein C-III (APOC3) is a key regulator of lipid metabolism. It inhibits the clearance of triglyceride (TG)-rich lipoproteins by hepatocytes, leading to elevated levels of TG and very-low-density lipoprotein (VLDL) in the bloodstream, as well as reduced high-density lipoprotein cholesterol (HDL-C). Due to its high specific expression in the liver, APOC3 has become an ideal target for RNA silencing technologies. The antisense oligonucleotide (ASO) therapy approved by the European Union and Arrowhead's RNAi therapy, ARO-APOC3, both target APOC3 by using RNA interference mechanisms to reduce its levels and thereby lower TG. ARO-APOC3 has shown remarkable performance in clinical trials, achieving over 90% reduction in TG level and significantly improving dyslipidemia. By silencing APOC3 gene expression, these therapies restore the normal lipoprotein clearance function of hepatocytes, providing a precise treatment strategy for diseases such as hypertriglyceridemia. The high efficiency of these therapies validates APOC3 as a drug target^101^.

### siRNA-based therapies

The mechanism of action of small interfering RNA (siRNA) is based on post-transcriptional gene silencing. siRNA molecules are typically specific and effective in knocking down disease-related genes^102^.

**HSD17B13.** 17-β-Hydroxysteroid Dehydrogenase 13 (HSD17B13) is a liver-specific lipid droplet protein that plays a key role in regulating hepatic lipid droplet homeostasis. Elevated expression of HSD17B13 promotes denovo lipogenesis and causes enlargement of lipid droplets, thereby contributing to the development of hepatic steatosis. Therefore, inhibiting the expression of HSD17B13 using oligonucleotide is a potential therapeutic strategy for MASLD^103^. Currently, RNAi therapies targeting HSD17B13 from Arrowhead and Alnylam are both being evaluated in Phase II clinical trials^104^, ^105^.

**SREBP-1c.** Sterol Regulatory Element-Binding Protein 1c (SREBP-1c) is a core transcription factor for lipid synthesis, driving the *de novo* synthesis of fatty acids and triglycerides in the liver by regulating genes such as fatty acid synthase (FAS) and acetyl-CoA carboxylase (ACC). Its overexpression is closely related to insulin resistance, hepatic steatosis, and inflammation, making it a key pathological mechanism in MASLD. In AML12 hepatocytes, SREBP-1c siRNA reduces lipid droplet formation and enhances fatty acid oxidation by upregulating PPARα^106^. siRNA targeting SREBP cleavage-activating protein (SCAP) (such as Merck's MK-0616) has significantly reduced LDL-C and triglycerides in a rhesus monkey model, indirectly supporting the therapeutic potential of targeting the SREBP pathway^107^.

**PLIN2.** Perilipin2 (PLIN2) is a key protein on the surface of lipid droplets (LDs). It promotes hepatic lipid storage by stabilizing the structure of lipid droplets and inhibiting the hydrolysis of triglycerides by lipases (such as ATGL)^108^. A team from Peking University has developed siRNA targeting PLIN2 and utilized a lipid nanoparticle (LNP) delivery system to achieve liver-specific silencing. In mouse models, this approach significantly alleviated steatosis without apparent toxicity. In a mouse model of fatty liver induced by a high-fat diet (HFD), PLIN2-siRNA downregulated PLIN2 expression, enhanced ATGL-mediated lipolysis, reduced hepatic triglyceride deposition, and improved insulin sensitivity^109^, ^110^.

**TGF-β1.** Transforming Growth Factor-β1 (TGF-β1) is a core molecule in the fibrotic pathway^111^. It promotes collagen deposition and extracellular matrix (ECM) remodeling by activating hepatic stellate cells (HSCs), leading to liver fibrosis and even cirrhosis. In HSC cell lines and mouse models, TGF-β1 siRNA inhibits the TGF-β1/Smad pathway, reduces the expression of fibrosis markers, and promotes HSC apoptosis. In a MASH model induced by a high-fat diet (HFD) combined with a choline-deficient and amino acid-defined diet (CDAA), TGF-β1 siRNA significantly alleviates hepatic steatosis, inflammation, and fibrosis^112^.

**RPL8.** Ribosomal Protein L8 (RPL8) is a key component of the large ribosomal subunit. By regulating ribosome biogenesis and translation efficiency, it affects the expression of genes involved in lipid synthesis. In HepG2 cells and high-fat diet (HFD) mice, si-RPL8 reduces lipid droplet formation, improves mitochondrial function, and decreases the expression of inflammatory factors (such as TNF-α and IL-6)^113^.

**TNF-α.** Tumor Necrosis Factor-α (TNF-α) is a pro-inflammatory cytokine that plays a central role in the progression of MASLD, contributing through activation of the inflammatory cascade, promoting hepatocyte apoptosis and fibrosis, and disrupting lipid metabolism. In a liver fibrosis model induced by bile duct ligation (BDL), TNF-α siRNA reduces collagen deposition (a 50% decrease in Sirius Red staining) and inhibits the expression of TGF-β1^114^-^116^.

### miRNA mimic or inhibitor-based therapies

**miR-122.** RG-101 is a GalNAc-conjugated anti-miR-122 oligonucleotide. Its Phase I trials showed strong efficacy: a single 2/4 mg/kg dose reduced viral load in 32 multi-genotype chronic HCV patients and modulated immune markers; a Phase I multicenter trial confirmed activity in multi-genotype, refractory/relapsed cases, alongside hepatic lipid-lowering effects and good tolerability^117^.

However, RG-101 development was halted in 2016: a Phase I jaundice case triggered an FDA hold. Despite Regulus submitting risk-mitigation plans, the agent was discontinued pre-Phase II and is no longer an active HCV candidate.

In MASLD, miR-122 is downregulated. Its mimic can reduce hepatic fat deposition and improve insulin resistance by inhibiting SREBP-1c^118^. Preclinical studies have shown that the miR-122 mimic Miravirsen can significantly reduce cholesterol and triglyceride levels^119^, ^120^.

**miR-29b.** GSK343 alleviates liver fibrosis by inhibiting the TGF-β/Smad signaling pathway, thereby reducing the activation of hepatic stellate cells and collagen deposition. Inhibition of the TGF-β pathway may upregulate the expression of miR-29b, which could further enhance the anti-fibrotic effect (further validation is needed)^121^. In models of insulin resistance, inhibition of miR-29b can enhance hepatic insulin sensitivity and reduce hepatic glucose output by activating the PI3K/Akt pathway^122^.

**miR-155.** TargomiRs utilize bacterial minicells to deliver miRNA mimics for gene regulation. Currently in the preclinical stage^123^, ^124^. The relevant inhibitors can decrease the expression of tumor necrosis factor α (TNF-α) and interleukin 6 (IL-6), thereby alleviating hepatic inflammation. In MASH models, inhibiting miR-155 can significantly improve liver injury and fibrosis^125^.

**miR-34a.** For example, the clinical trial NCT04053549 evaluates its efficacy in MASH. Phase: This trial is a Phase I/II study, aiming to assess the safety, tolerability, and preliminary efficacy of miR-34a inhibitors in patients with MASH^126^. Intervention: Antisense oligonucleotides (ASO) or small interfering RNA (siRNA) are used to target and inhibit miR-34a, with enhanced therapeutic effects achieved through liver-targeted delivery systems (such as lipid nanoparticles or ASGPR ligand conjugation)^127^.

## Safety issues of synthetic oligonucleotides

Synthetic oligonucleotides have a wide range of applications in the biomedical field, however, their use is associated with certain safety concerns, which are mainly reflected in the following aspects:

### Off-target effects

Off target effects can occur due to non-specific binding of oligonucleotides to non-target RNA, or unintended gene silencing triggered by seed region matching^128^. For instance, Mipomersen, an ASO targeting ApoB, consists of a phosphorothioate backbone that can bind non-specifically to ApoB mRNA, potentially leading to off-target interactions and resulting in elevated liver enzyme levels^51^, ^129^-^131^.

### Immunogenicity

Synthetic oligonucleotides may be recognized by the immune system as foreign substances, thereby triggering innate immune responses, such as the activation of Toll-like receptors (TLRs) which leads to the release of inflammatory cytokines^132^. For example, DNA containing unmethylated CpG motifs can activate TLR9, triggering a cytokine storm. Immunogenicity of PS-modified ASO: The phosphorothioate (PS) backbone activates TLR7/8, leading to elevated liver enzymes and systemic inflammatory responses in MASLD patients, as demonstrated in clinical studies^51^.

### Toxicity

Certain chemical modifications (such as phosphorothioate) may lead to kidney or liver toxicity, and long-term administration may accumulate adverse effects. For example, the PS-ASO drug Oligomycin has been shown to induce acute kidney injury in mouse models, characterized by elevated serum creatinine and tubular necrosis^133^. High doses of ASO may disrupt normal RNA metabolism or cause cholestasis^134^.

### Delivery systems

The ASGPR receptor, which is highly expressed in the liver (such as galactose-modified lipid nanoparticles), provides enhanced targeting for drug-delivery systems, but excessive delivery may exacerbate liver injury. GalNAc-siRNA (such as Fesomersen) has been associated with dose-dependent elevation in liver enzymes in patients with MASH^135^. The inflammatory state within the liver of MASLD patients may enhance the immunogenicity of AAV capsid proteins^136^, ^137^. During AAV8 vector-based treatment for MASH, pre-existing antibodies can lead to reduced therapeutic efficacy^138^.

## Challenges and future directions

In recent years, a deeper understanding of the pathological mechanism underlying MASLD has propelled the development of emerging oligonucleotide therapies, such as antisense oligonucleotides and small interfering RNAs. Meanwhile, liver-targeted and hepatocyte-specific gene editing technologies-including CRISPR systems (delivered via AAV or Lipid Nanoparticles, LNP)-are advancing rapidly in both basic research and clinical translation. These innovative approaches offer the potential for precise modulation of gene expression involved in lipid metabolism, inflammation, and fibrosis, thereby addressing limitations of current therapies and paving the way for personalized, mechanism-based interventions to halt or reverse disease progression. Despite these promising advances, challenges remain in optimizing liver-targeted delivery efficiency, ensuring long-term safety, and tailoring precise application to different pathological stages. The multifactorial nature of MASLD, involving metabolic, inflammatory, and fibrotic pathways, further complicates the development of effective treatments and underscores the need for personalized approaches. Additionally, early diagnosis remains difficult, often resulting in delayed intervention and progression to advanced liver disease. Future research should focus on elucidating the molecular mechanisms underlying MASLD to identify novel therapeutic targets. Future efforts are needed to optimize delivery systems, develop of multi-target combination therapies, and design precise drugs based on the individual pathological characteristics of MASLD. These advances are expected to facilitate the translation of oligonucleotide therapies from basic research to clinical application, offering new therapeutic hope for the growing global population affected by MASLD.

## Figures and Tables

**Figure 1:**
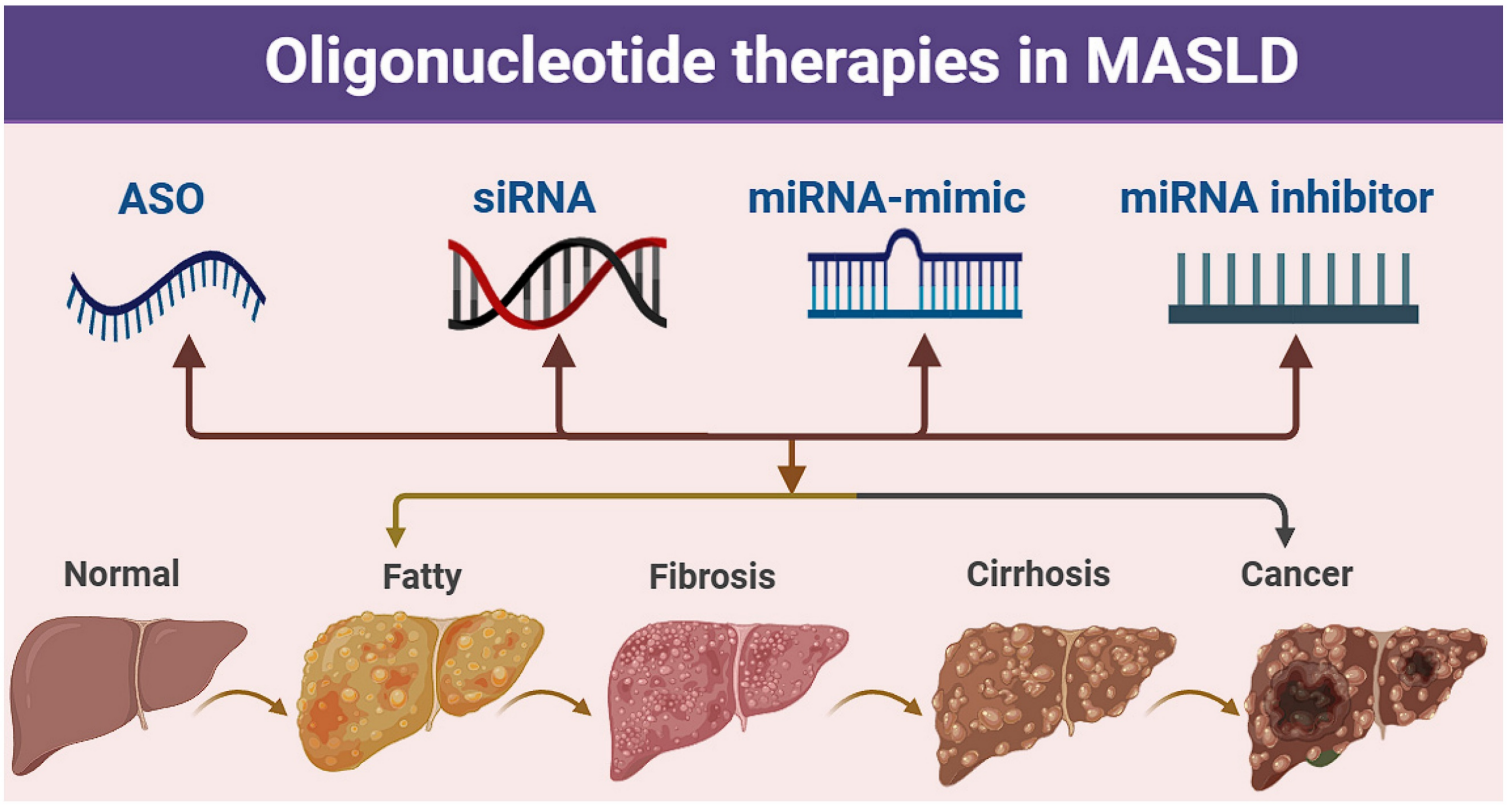
** Oligonucleotide-related therapeutic strategies for MASLD.** Pathological progression of the liver from the normal state to fatty liver, fibrosis, cirrhosis, and cancer, along with the potential intervention strategies of antisense oligonucleotide (ASO), small interfering RNA (siRNA), miRNA mimic, and miRNA inhibitor.

**Figure 3:**
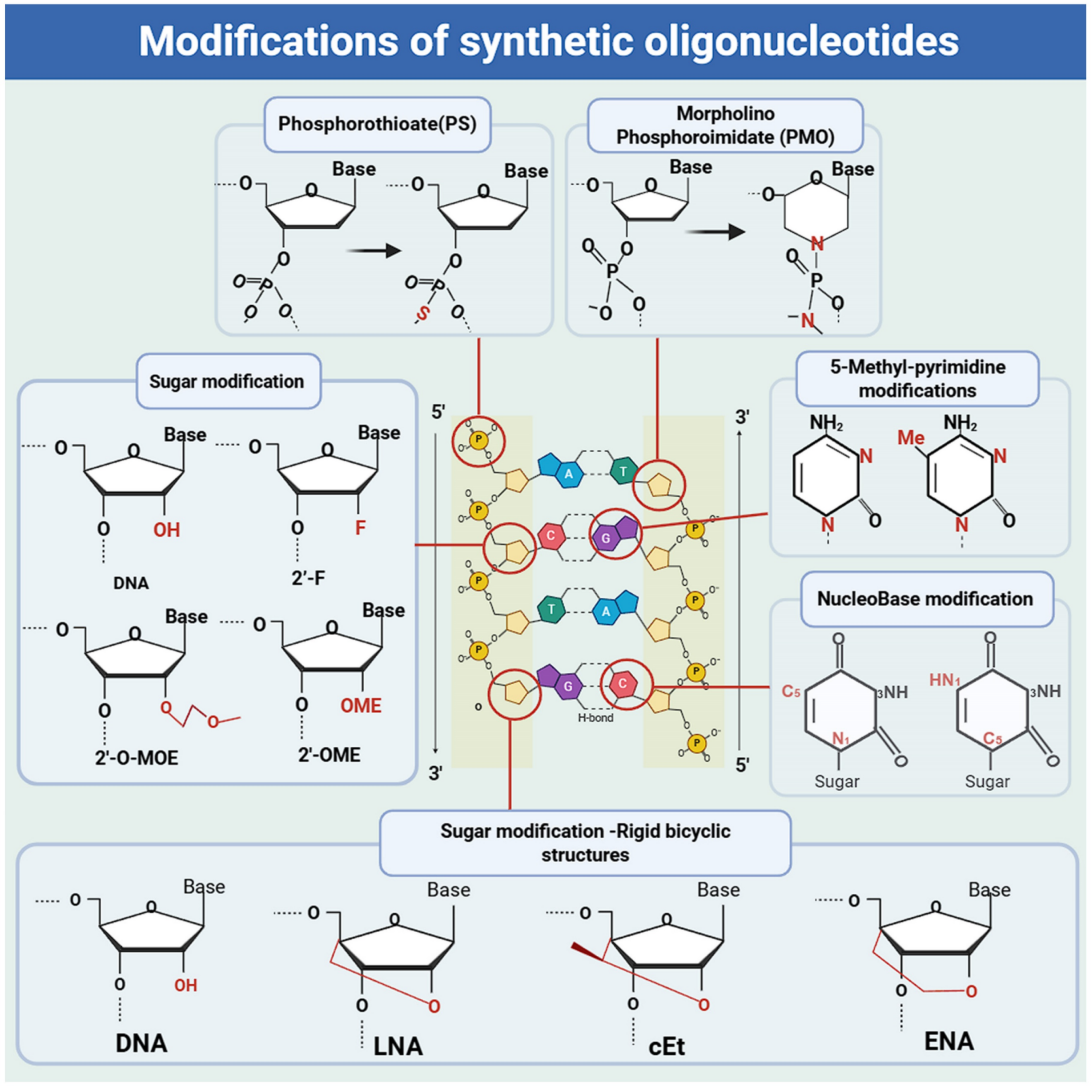
** Common chemical modifications for improving pharmacodynamic and pharmacokinetic properties in approved therapeutics**. Schematic of an RNA nucleotide and its chemical modification sites (backbone, nucleobase, 2'-ribose substitutions and Rigid bicyclic sugar modifications). Backbone modifications: PS (phosphorothioate), PMO (phosphorodiamidate morpholino oligonucleotide). Nucleobase modifications: 5'-methyl-pyrimidine modifications; nucleobase modifications. 2'-ribose substitutions modifications: 2'-substitutions (2'-F/2'-fluoro, 2'-MOE/2'-O-methoxyethyl, 2'-OMe/2'-O-methyl). Rigid bicyclic sugar modifications: LNA (locked nucleic acid), cEt (constrained ethyl bridged nucleic acid), ENA (ethylene-bridged nucleic acid).

**Figure 4:**
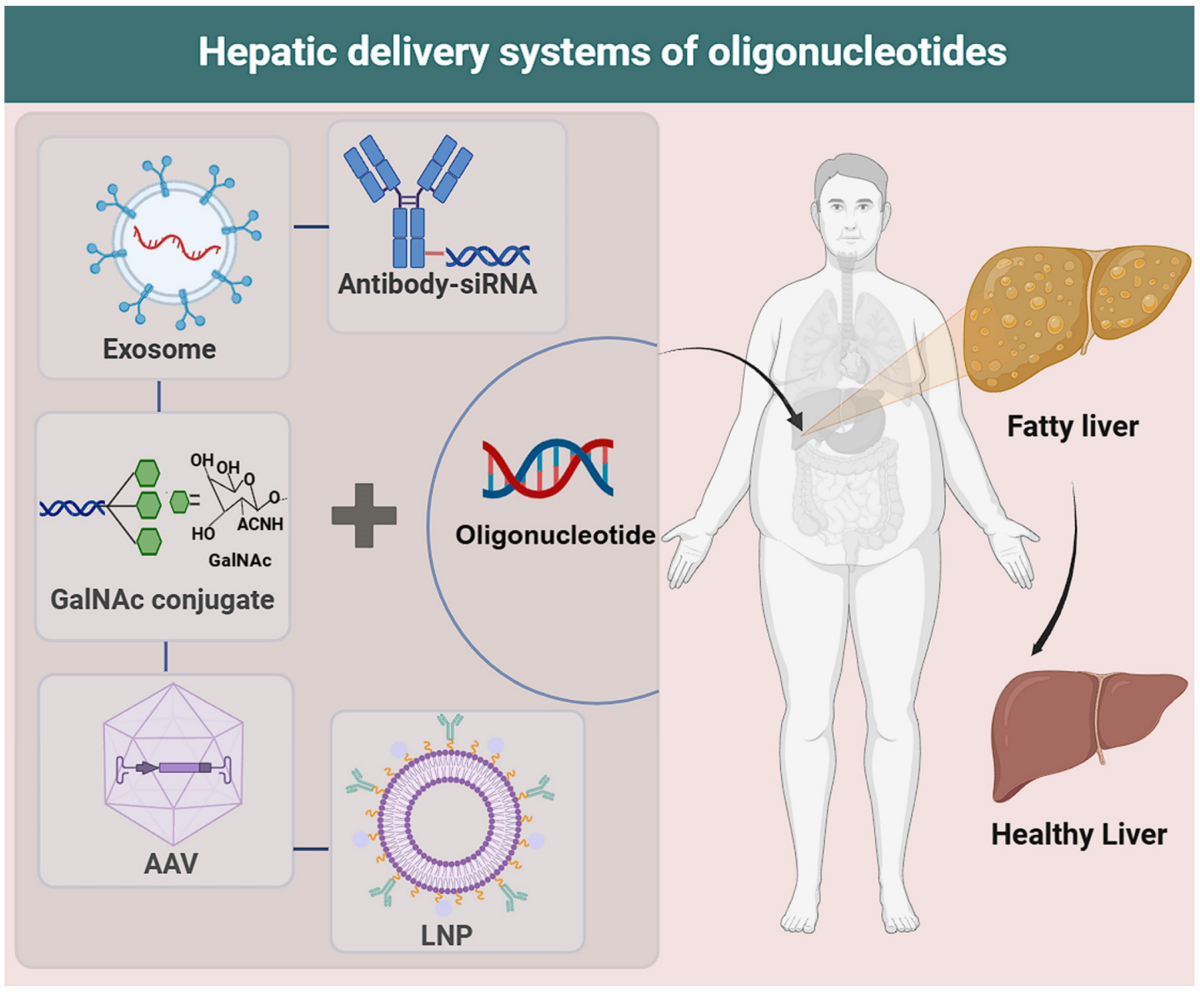
**Delivery technologies for oligonucleotides.** LNP: Composed of ionizable lipids, cholesterol, phospholipids, and polyethylene glycol (PEG), which collectively mediate its function. AAV: Enters target cells via endocytosis (capsid recognizes specific surface receptors); releases single-stranded DNA (after capsid disassembly) that is repaired to double-stranded DNA, then enters the nucleus for exogenous gene delivery. GalNAc conjugate: Synthetic triantennary N-acetyl-d-galactosamine (GalNAc) conjugated to siRNA, mediating binding to hepatocyte surface asialoglycoprotein receptor (ASGPR). Exosome: Has a lipid bilayer membrane and internal lumen; lumen-loaded functional molecules (nucleic acids: siRNA/mRNA; small-molecule drugs: chemotherapeutics; proteins: antibodies/enzymes) are core for therapeutic effects. Antibody-siRNA: Novel targeted therapy (antibody/fragment conjugated to siRNA via linkers); antibody guides siRNA to specific cells, where siRNA silences disease genes for targeted gene silencing.

**Figure 5:**
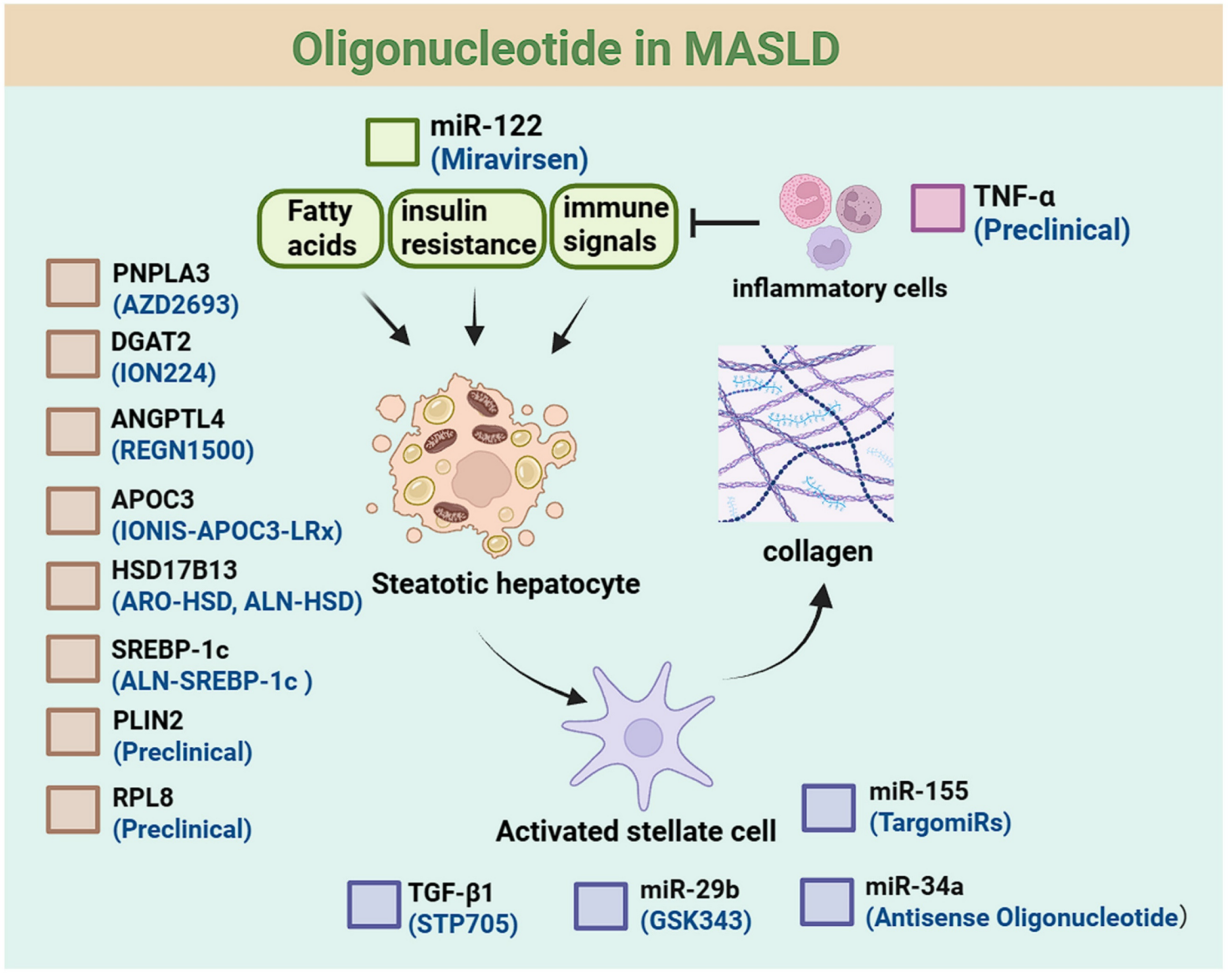
** Oligonucleotide drugs for MASLD.** This schematic outlines the pathophysiological regulation of steatotic hepatocytes, including effects of upstream factors (fatty acids, insulin resistance, immune signals). It also depicts activated stellate cell-collagen links and inflammatory cell involvement, with key regulatory molecules labeled. Available therapeutic drugs for each target are listed below the targets.

**Table 1:** The advantages and disadvantages of each type of oligonucleotide.

Oligonucleotide	Advantages	Disadvantages
ASO^40^, ^41^	Targets mRNA/pre-mRNA via multiple mechanisms.	Cellular uptake relies on delivery vectors; homologous sequences risk off-target effects.
siRNA^42^, ^43^	High mRNA silencing efficiency via RNAi pathway; strong sequence specificity.	Rapid degradation by nucleases; short *in vivo* half-life.
miRNA-mimic^44^	Mimics endogenous miRNAs to co-regulate multiple target genes.	Multi-target regulation may disrupt normal gene regulatory networks.
miRNA-inhibitor^45^	Specifically blocks pathologically overexpressed miRNAs.	May interfere with physiological functions of normal miRNAs.
Aptamer^46^, ^47^	Low immunogenicity; excellent biocompatibility.	Limited penetration in solid tumors.
Gapmer^48^	Higher silencing efficacy than traditional ASOs.	High risk of off-target RNA cleavage.
saRNA^37^, ^49^	Self-replicates for long-term protein production.	Large molecular size/negative charge leads to high delivery difficulty.
SSO^16^	Targets pre-mRNA splicing to reprogram isoforms; high splicing-element specificity.	Delivery-dependent uptake; risks disrupting physiological pre-mRNA splicing.

**Table 2:** The viral vector delivery systems for oligonucleotides.

Vector Type	Advantages	Disadvantages	Application Potential in MASLD
Adeno-associated Virus (AAV)	Low immunogenicity, long-term expression, strong targeting ability.	Small capacity (< 5 kb), high production cost.	Deliver anti-fibrosis genes (such as miR-296 mimetics).
Lentivirus (LV)	Integrate into the genome, efficiently transduce dividing/non-dividing cells.	Risk of insertional mutagenesis, high immunogenicity.	Gene editing (such as CRISPR-Cas9).
Adenovirus (AdV)	High transduction efficiency, large capacity (> 30 kb).	High immunogenicity, transient expression.	Short-term gene delivery (such as inflammatory regulatory factors).

**Table 3:** Therapeutic advantages of exosomes in the treatment of MASLD.

Characteristics	Therapeutic Advantages in MASLD Treatment
Natural carrier	Derived from cells, low immunogenicity, high biocompatibility.
Barrier-crossing ability	Can cross the blood-brain barrier (BBB), placental barrier, and target the liver (via ASGPR and other receptors).
Cargo diversity	Load mRNAs, siRNAs, proteins, drugs, etc., to regulate multiple pathways (such as inflammation, fibrosis, metabolism).
Targetability engineering	Enhance liver-specific targeting through surface modification (such as antibodies, ligands).
Stability	The outer membrane protects the cargo, prolonging the in-vivo circulation time (compared to nucleic acid drugs).

**Table 4:** Hepatic delivery systems for oligonucleotides in MASLD.

Delivery System	Core Advantages	Key Disadvantages	MASLD Application Highlights
Cationic liposomes	High loading efficiency (>90%), simple preparation, modifiable for targeting.	Poor *in vivo* stability, rapid RES clearance, limited targeting specificity.	Potential anti-inflammatory delivery (targeting TNF-α)^82^.
Lipid nanoparticles	High encapsulation efficiency (>85%), pH-responsive release, targetable to hepatocytes/macrophages, 30-fold dose reduction.	Immunogenicity risk, complex manufacturing, high cost.	Loaded with HMGB1/Bid-siRNA to improve MASH inflammation/fibrosis^83^, ^84^.
GalNAc conjugates	Exceptional hepatocyte specificity, 10-100-fold potency enhancement, low immunogenicity, clinically validated safety.	Hepatocyte-only targeting, requires chemical modification, receptor saturation at high doses.	Targeting Stk25/MCJ to improve steatosis/fibrosis^85^.
Viral vector platforms	Ultra-high transfection efficiency, long-term stable expression, large payload capacity.	High immunogenicity, insertional mutagenesis risk, scalability challenges.	Efficacious in liver fibrosis models (targeting PAI-1/TIMP-1)^86^.
Exosomes	Excellent biocompatibility, low immunogenicity, co-loadable with multiple molecules.	Low yield, complex purification, limited loading capacity.	Loaded with TNF-α ASO to improve MASH and alleviate liver fibrosis^87^, ^88^.
Antibody-Drug Conjugates	Precise targeting of specific hepatic cell populations, minimal off-target effects, low toxicity.	Complex preparation, extremely high cost, efficacy affected by antigen heterogeneity.	Targeting CD248/F4/80 for anti-fibrosis/anti-inflammation^89^, ^90^.

**Table 5:** Application of ASO in metabolic dysfunction-associated steatohepatitis.

Target	Pathway	Mechanism of Action	Preclinical/Clinical Status	Drug/Developer Company
PNPLA3	Lipid metabolism/Inflammation.	Inhibit lipolysis and reduce intrahepatic triglyceride accumulation.	Phase I Trial	AZD2693 (AstraZeneca)
DGAT2	Lipid synthesis.	Block triglyceride synthesis and promote fatty acid oxidation.	Phase II Trial	ION224 (Ionis Pharmaceuticals)
ANGPTL4	Lipoprotein metabolism/Inflammation.	Enhance lipoprotein lipase activity and reduce triglyceride levels.	Phase II Trial (REGN1500, Regeneron)	REGN1500 (Regeneron)
APOC3	Triglyceride metabolism.	Inhibit the secretion and clearance of triglyceride-rich lipoproteins.	Phase III Trial (IONIS-APOC3-LRx, Ionis)	IONIS-APOC3-LRx (Ionis Pharmaceuticals)

**Table 6:** Application of siRNA in metabolic dysfunction-associated steatohepatitis.

Target	Pathway		Mechanism of Action	Preclinical/Clinical Status	Drug/Developer Company
HSD17B13	Lipid metabolism/Oxidative stress.		Inhibit 17β-hydroxysteroid dehydrogenase activity, reduce lipid synthesis and oxidative stress.	Phase II Trial	ARO-HSD (Arrowhead Pharmaceuticals); ALN-HSD (Alnylam Pharmaceuticals; Regeneron)
SREBP-1c	Lipid synthesis.		Reduce the expression of fatty acid synthase (FAS) and acetyl-CoA carboxylase (ACC).	Phase II Trial	ALN-SREBP (Alnylam Pharmaceuticals)
PLIN2	Lipid droplet stability.		Enhance the secretion of very low-density lipoprotein (VLDL), promote lipid mobilization.	Preclinical	Not public (Developed by a Chinese team)
TGF-β1		Fibrosis.	Inhibit the activation of hepatic stellate cells (HSC), reduce collagen deposition.	Phase II Trial	STP705 (Sirnaomics)
RPL8		Ribosome function.	Reduce ribosome-mediated lipid synthesis, alleviate endoplasmic reticulum (ER) stress.	Preclinical	Not public (Developed by the Chinese Academy of Sciences)
TNF-α		Inflammation.	Block NF-κB signaling pathway, inhibit the secretion of pro-inflammatory factors.	Preclinical	Not public (Early-stage development by some companies)
					

**Table 7:** miRNA mimic or inhibitor-based therapies.

Target	Drug Name	Mechanism of Action	Clinical Trial Phase	Indication	Delivery System
miR-122	RG-101	Inhibit SREBP-1c-mediated lipid synthesis and improve insulin resistance.	Discontinued	MASLD (Hepatic Steatosis)	GalNAc-conjugated (ASO)
miR-122	Miravirsen	Preclinical, downregulate SREBP-1c, SCD-1, reduce cholesterol and triglyceride.	Preclinical	Hypercholesterolemia/MASLD	GalNAc-conjugated (ASO)
miR-29b	GSK343	Inhibit the TGF-β/Smad pathway, reduce HSC activation and collagen deposition (indirectly upregulate miR-29b).	Phase I/II (Ongoing)	MASH-related Fibrosis	Lipid Nanoparticle (LNP)
miR-155	TargomiRs	Inhibitor, downregulate TNF-α, IL-6 expression and alleviate inflammation.	Preclinical	MASH-related Inflammation	Exosome/Polymer Nanoparticle
miR-34a	Antisense Oligonucleotide	Inhibitor, block miR-34a via ASO/siRNA, activate PPARγ and improve lipid metabolism.	Phase I/II (Ongoing)	MASH (Fibrosis)	LNP/ASGPR Ligand-conjugated
